# ^1^H-NMR-Based Metabonomic Studies on the Anti-Depressant Effect of Genipin in the Chronic Unpredictable Mild Stress Rat Model

**DOI:** 10.1371/journal.pone.0075721

**Published:** 2013-09-18

**Authors:** Jun-Sheng Tian, Bi-Yun Shi, Huan Xiang, Shan Gao, Xue-Mei Qin, Guan-Hua Du

**Affiliations:** 1 Modern Research Center for Traditional Chinese Medicine of Shanxi University, Taiyuan, P. R. China; 2 Physical Education Departments of Shanxi University, Taiyuan, P. R. China; 3 Research Center of Traditional Chinese Medicine, Tianjin University of Traditional Chinese Medicine, Tianjin, P. R. China; 4 Institute of Materia Medica, Chinese Academy of Medical Sciences & Peking Union Medical College, Beijing, P. R. China; McLean Hospital/Harvard Medical School, United States of America

## Abstract

The purpose of this work was to investigate the anti-depressant effect of genipin and its mechanisms using ^1^H-NMR spectroscopy and multivariate data analysis on a chronic unpredictable mild stress (CUMS) rat model. Rat serum and urine were analyzed by nuclear magnetic resonance (NMR)-based metabonomics after oral administration of either genipin or saline for 2 weeks. Significant differences in the metabolic profile of the CUMS-treated group and the control group were observed, which were consistent with the results of behavioral tests. Metabolic effects of CUMS included decreases in serum trimetlylamine oxide (TMAO) and β-hydroxybutyric acid (β-HB), and increases in lipid, lactate, alanine and N-acetyl-glycoproteins. In urine, decreases in creatinine and betaine were observed, while citrate, trimethylamine (TMA) and dimethylamine were increased. These changes suggest that depression may be associated with gut microbes, energy metabolism and glycometabolism. Genipin showed the best anti-depressive effects at a dose of 100 mg/kg in rats. These results indicate that metabonomic approaches could be powerful tools for the investigation of the biochemical changes in pathological conditions or drug treatment.

## Introduction

**Figure 1:**
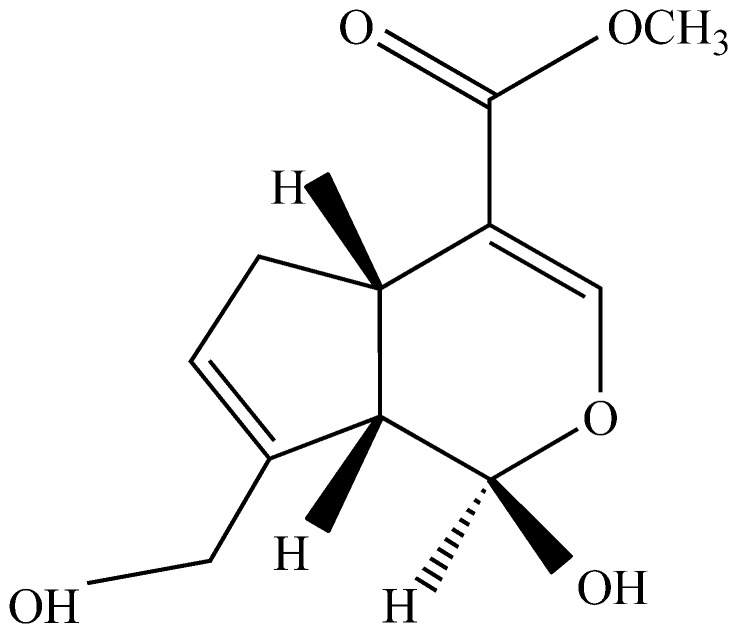
Structure of Genipin.

Genipin (Figure 1), the aglycone of geniposide, is one of the key bioactive constituents extracted from the fruit of *Gardenia jasminoides* E. (Chinese herbal name Zhi Zi). It is one of the commonly used herbal medicines or functional food supplements in China and other oriental countries. In the food industry, genipin is used to produce natural colorants (blue, green, violet, etc.) after simple reactions with primary amines [1], [2]. In the medicinal field, as a bioactive agent, genipin has drawn attention for its anti-inflammatory [3], antibacterial, antithrombotic [4], hepatic-protective and choleretic effects [5]–[7].

Pharmacokinetics studies have suggested that geniposide, when orally administered, is hydrolyzed into genipin by enzymes produced by intestinal bacteria [8]. It is genipin, not geniposide, which functions as the main bioactive compound to cause the pharmacological activities of gardenia [9].

Depression is a common and persistent psychiatric illness and presents a considerable social and economic burden [10]. The World Health Organization (WHO) predicted that depression would be the first leading cause of death worldwide by 2030. Chemical anti-depressant drugs include Tricyclic antidepressants (TCAs), monoamine oxidase inhibitors (MAIOs), and selective serotonin reuptake inhibitor (SSRIs), such as Amitriptyline, Fluoxetine, Paroxetine, Venlafaxine, etc. However, most of these drugs are associated with side effects, which are far from satisfaction for patients.

The CUMS model is a well-validated and widely used rodent model of depression [11], [12]. In CUMS, animals are exposed to different kinds of stresses that mimic the stresses in human life [13].

Metabonomics is a rapidly emerging area of “-omics” research in which endogenous metabolites of biofluids (such as serum and urine) or tissues (liver and brain, etc.) are comprehensively assessed, followed by systematic identification and accurate quantification [14]. With the development of effective analytical technologies and methods, metabonomic approaches are gaining widespread use for the mechanistic study of diseases [15] or potential biomarker discovery [16]. Metabonomic information is obtained by maximum data capture from samples using Nuclear Magnetic Resonance (NMR), Mass Spectrometry (MS), High-Performance Liquid Chromatography (HPLC) and Gas Chromatography (GC) followed by multivariate data analysis. ^1^H-NMR-based metabonomics have been suggested as an attractive tool for the investigation of the effects of depression [17], [18] due to its non-destructive nature and rapid collection of data.

Multivariate data analysis is employed to discriminate samples by comparing metabolites and by visualization of clustering between different groups [19]. Principal component analysis (PCA), partial least squares-discriminate analysis (PLS-DA) and orthogonal-projection to latent structure-discriminate analysis (O-PLS-DA) are the primary types of multivariate data analysis utilized [20]. Metabonomic approaches are becoming more common in fields such as animal husbandry, food chemistry, plant genetic engineering technology, pathology of diseases and others [21], [22].

The anti-depressant-like effect of genipin in forced swimming test (FST) and tail suspension test (TST) has been questioned [23], [24]. However, little has been published on the response of rat biological systems to the intake of genipin. To confirm the antidepressant-like effect of genipin and to explore the possible pharmacological mechanisms in the rat CUMS model, we measured physiological and behavioral outcomes such as body weight, sucrose preference and locomotor activity. ^1^H-NMR-based metabonomic strategies were applied to analyze the endogenous metabolites in rat serum and urine. The potential biomarkers involved in the therapeutic effects of genipin were investigated in this study.

## Materials and Methods

### Animals and reagents

Male Sprague Dawley rats, weighing 180–200 g, were purchased from Beijing Vital River Laboratories Co. (SCXK (Jing) 2011–0012). The animals were acclimated to the new environment for 1 week before experiments were performed. All animals were housed 6 per cage under controlled conditions of light (12-h light/dark cycle, lights on at 8:00 a.m.), temperature (24±1°C) and humidity (45±15%) with free access to food and water, except when they were subjected to deprivation stressors during the CUMS procedure. This study was carried out in strict accordance with the recommendations in the Guide for the Care and Use of Laboratory Animals of the National Institutes of Health. All experimental procedures were approved by the Committee on the Ethics of Animal Experiments of Shanxi University. The animals were sacrificed after the anesthesia by ethyl carbamate injection; the maximum efforts were made to minimize suffering, and minimum number of animals necessary for the collection of reliable data was used.

Genipin was extracted and hydrolyzed from the fruit of *Gardenia jasminoides* E. and measured to be 98% pure. The genipin was supplied by Lin-chuan-zhi-xin Bio-Technology Co. in Jiangxi Province of China. Venlafaxine hydrochloride capsules (Chengdu Kanghong Pharmaceutical, Lot No. 090705) were commercially obtained from the First Hospital of Shanxi Medical University, Taiyuan, China). All other chemical reagents were analytical grade.

### Treatments

Animals were weighed and randomly divided into 6 groups: a control group not receiving CUMS (NS), a group receiving CUMS and vehicle (MS) and five groups receiving CUMS and a test compound: Venlafaxine group (50 mg/kg body weight, positive group, YV), genipin high dose group (100 mg/kg body weight, GH), genipin middle dose group (50 mg/kg body weight, GM) and genipin low dose group (25 mg/kg body weight, GL). The CUMS procedures were conducted for 4 weeks on all animals except for those of NS group, while drug administration was carried out daily in the last 2 weeks. Genipin and Venlafaxine were dissolved with saline to the required concentration and administered to the CUMS-treated rats via gastric intubation for 2 weeks. The NS and MS groups were given normal saline at the same volume. All drugs were administrated 30 min before exposure to stresses.

### CUMS procedure and sample collection

The control rats were housed together, while the CUMS-treated rats were housed individually. The procedure was performed as described previously [25] with minor modifications. The CUMS-treated rats were subjected to the following stressors in a random order every day for 4 weeks: 24 h food deprivation, 24 h water deprivation, exposure to an experimental room at 45°C for 5 min, swimming in 4°C cold water for 5 min, tail clamp for 2 min, foot-shock for 2 min, noises for 3 h. After the last stressor was performed on the 28^th^ day, urine was collected continuously (12 h) in metabolism cages. Following urine collection, all animals were anesthetized by ethyl carbamate injection (1.5 g/kg body weight), and blood samples were acquired via the arteria cruralis. Both blood and urine were centrifuged at 4°C at 3,000 r/min for 15 min, and the supernatants were stored at −80°C prior to use.

### Behavioral tests

#### Body weight

The body weight of rats on days 0, 7, 14, 21 and 28 was measured.

#### Sucrose Preference Test

Before the experiment was carried out, all rats were exposed to a 1% sucrose solution for 24 h to avoid neophobia. Then, two bottles, one containing 1% sucrose solution and the other containing tap water, were weighed and presented to each rat for 4 h. The position of the tap water bottle and sucrose solution bottle were randomly determined. Sucrose and water consumptions (g) were measured, and the sucrose preference was calculated using the equation: 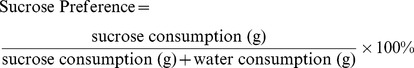



#### Open-field test

The open-field test (OFT) was performed according to a previous study [26] once a week. The custom-made apparatus was a 100×100×40 cm^3^ black metal cage whose bottom was divided into 25 equal sectors by white stripes. Each rat was gently placed in the central square and observed for 5 min. The number of crossings and rearings during the last 4 min was recorded.

### ^1^H-NMR metabonomics of serum and urine

#### Samples preparation

Serum samples were prepared as follows: 450 µL serum and 350 µL D2O (Deuterium Oxide) were pipetted individually and mixed together. After centrifugation at 4°C and 13,000 r/min for 20 min, 600 µL of the supernatants were transferred into 5 mm NMR tubes for 1H-NMR analysis. Urine samples were thawed prior to use. A total of 500 µL of each sample was diluted with 200 µL of phosphate buffer (0.2 M Na2HPO4/NaH2PO4, pH 7.4) containing D2O for the purpose of field lock and 0.03% sodium 3-trimethylsilyl-(2, 2, 3, 3-2H4)-1-propionate (TSP) as a chemical shift reference. The whole mixture was centrifuged at 4°C and 13,000 r/min for 20 min, and aliquots of 600 µL were transferred into 5 mm NMR tubes for the 1H-NMR analysis.

#### 1H-NMR Spectrometry

1H-NMR spectra of the serum and urine samples were recorded at 300 K on a Bruker 600-MHz AVANCE III NMR spectrometer (Bruker, Germany), operating at 600.13 MHz for 1H. Samples were analyzed using one-dimensional Carr-Purcell-Meibom-Gill (CPMG) NMR spectra (for serum) and Nuclear Overhauser Effect Spectroscopy (NOESY, RD-90°-t1-90°-tm-90°-acquire) NMR spectra (for urine) with water suppression. A total spin-spin relaxation delay (2nτ) of 320 ms was used and a total of 64 transients were collected for the spectra.

### Data analysis

Results of the behavioral data were expressed as mean±S.E.M. Statistical differences were determined using an independent-sample *t*-test by SPSS 17.0. Values of *p*<0.05 was considered as statistically significant difference.

The ^1^H-NMR spectra were processed using MestReNova software (Mestrelab Research, Santiago de Compostella, Spain). All the spectra were manually corrected for phase and baseline distortions. The spectra of serum were referenced internally to the chemical shift of creatinine at δ 3.03 ppm. The serum spectra were divided and the signal integral computed in 0.01 ppm intervals across the region δ 0.50-9.00 ppm. The region of δ 4.67-5.22 ppm was removed to eliminate the effects of imperfect water saturation. The data was then normalized to the total sum of the spectra prior to analysis. The spectra of urine were referenced to the chemical shift of TSP at δ 0.00 ppm. The spectra were divided and the signal integral computed in 0.01 ppm intervals across the region δ 0.50-9.00 ppm. The regions of δ 4.70–5.10 ppm and δ 5.55–6.15 ppm were removed to eliminate the influence of water and urea.

Multivariate statistical analysis (PCA) was performed to process the acquired NMR data using SIMCA-P 13.0 (Umetric, Sweden). In PCA, samples from different groups were classified. The results were presented in the form of score plots, where each point represents an individual sample (to show the group clusters), and loading plots, where each coordinate represents one ^1^H-NMR spectral region (to identify the variables contributing to the classification). According to the criterion of loading plots, the further the plot sits from the origin, the greater the contribution of the metabolite to the separation of the groups involved. Each of the metabolites was identified, and the variables that demonstrated large contributions to the CUMS-induced depression or to the anti-depressive effects of drug treatment were selected as major metabolites. An independent sample *t-*test was further used to investigate alterations in endogenous metabolites using SPSS 17.0 (Chicago, IL, USA). *p*<0.05 was considered statistically significant difference.

## Results and Discussion

### Effects of Behavioral Tests in CUMS-treated rats

#### Body weight

**Figure 2:**
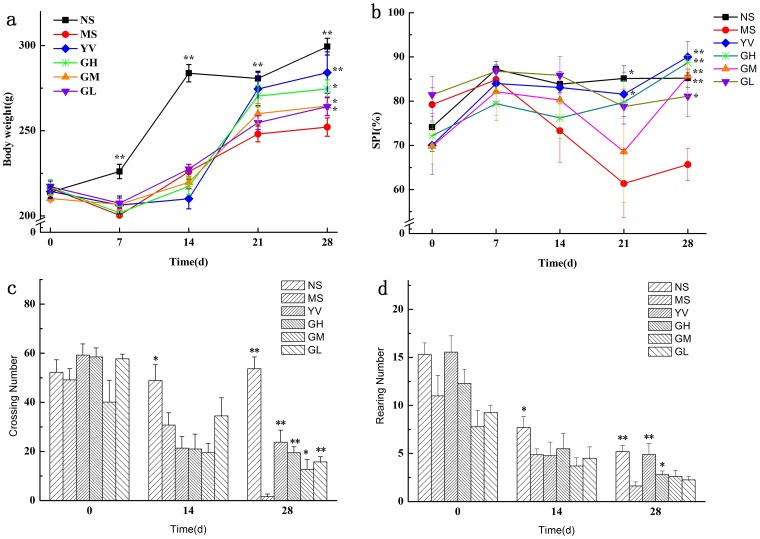
Effect of genipin treatment on the body weight (a), sucrose preference (b), crossing numbers (c), and rearing numbers (d) in CUMS exposed rats (NS: control group not receiving CUMS, MS: CUMS and vehicle, YV: CUMS plus Venlafaxine, GH: CUMS plus genipin high dose, GM: CUMS plus genipin middle dose, GL: CUMS plus genipin low dose). Values given are the mean±SEM (n = 10). **p<0.01, *p<0.05 as compared with the model.

As shown in Figure 2a, the body weight of MS group rats decreased, while that of the control group increased (*p*<0.05) in the first week of the CUMS procedure. This result demonstrates that the rats were suffering from the unpredicted stresses and eating less food than normal ones during the first stressful week. During the second week, the body weight of the MS group rats began to increase slowly, while the weight of the NS group rats increased dramatically. After 2 weeks' drug administration, body weight increased in the YV, GM, and GL groups significantly (*p*<0.05), and the weight of the GH group was significantly (*p*<0.01) higher than that of the MS group. Treatment with genipin increased the body weight of CUMS-treated rats, indicating an improvement in appetite and emotion.

#### Sucrose preference

The sucrose preference test was used to determine the animals' depressive state. As shown in Figure 2b, the baseline sucrose preference index was about 74%, and significant differences were observed between the NS and MS groups at the end of the third and fourth week. During the last two weeks, the sucrose preference was sustained at about 61% in the MS group, indicating a clear depressive state. After the administration of Venlafaxine or genipin for two weeks, the sucrose preferences of the YV, GH, GM (*p*<0.01) and GL (*p*<0.05) groups were significantly higher than the MS group. Treatment with genipin increased the sucrose preference and prevented anhedonia, which is the key indicator of successful implementation of the CUMS model.

#### Open Field Test

The OFT is commonly used to investigate locomotor activity, exploratory and depressive-like behaviors in experimental animals [27]. The number of crossings and rearings was measured during the OFT. Figure 2c and Figure 2d showed the numbers of crossings and rearings before, during and after the CUMS procedure. After four weeks, the number of crossings and rearings in the MS group was significantly decreased (*p*<0.01) compared with NS group. This decrease suggested that locomotor activity was seriously affected in the CUMS-treated rats. After two weeks of drug administration, both the crossings and rearings were significantly increased compared with the MS group (*p*<0.01). Of the three doses of genipin, the GH group showed the best effect on increasing animals' locomotor activities.

The CUMS procedure was used to mimic the stresses of daily human life in an animal model. This procedure induces anhedonia, which is a core symptom of depression in rats. The ability of the animals to maintain body weight, the sucrose preference test and the open field test are all evaluations of whether the depressive animal model was duplicated successfully. In this work, the CUMS procedure was very effective in duplicating the behavioral results indicative of depressive state.

### Analysis of ^1^H-NMR Profiles

**Figure 3:**
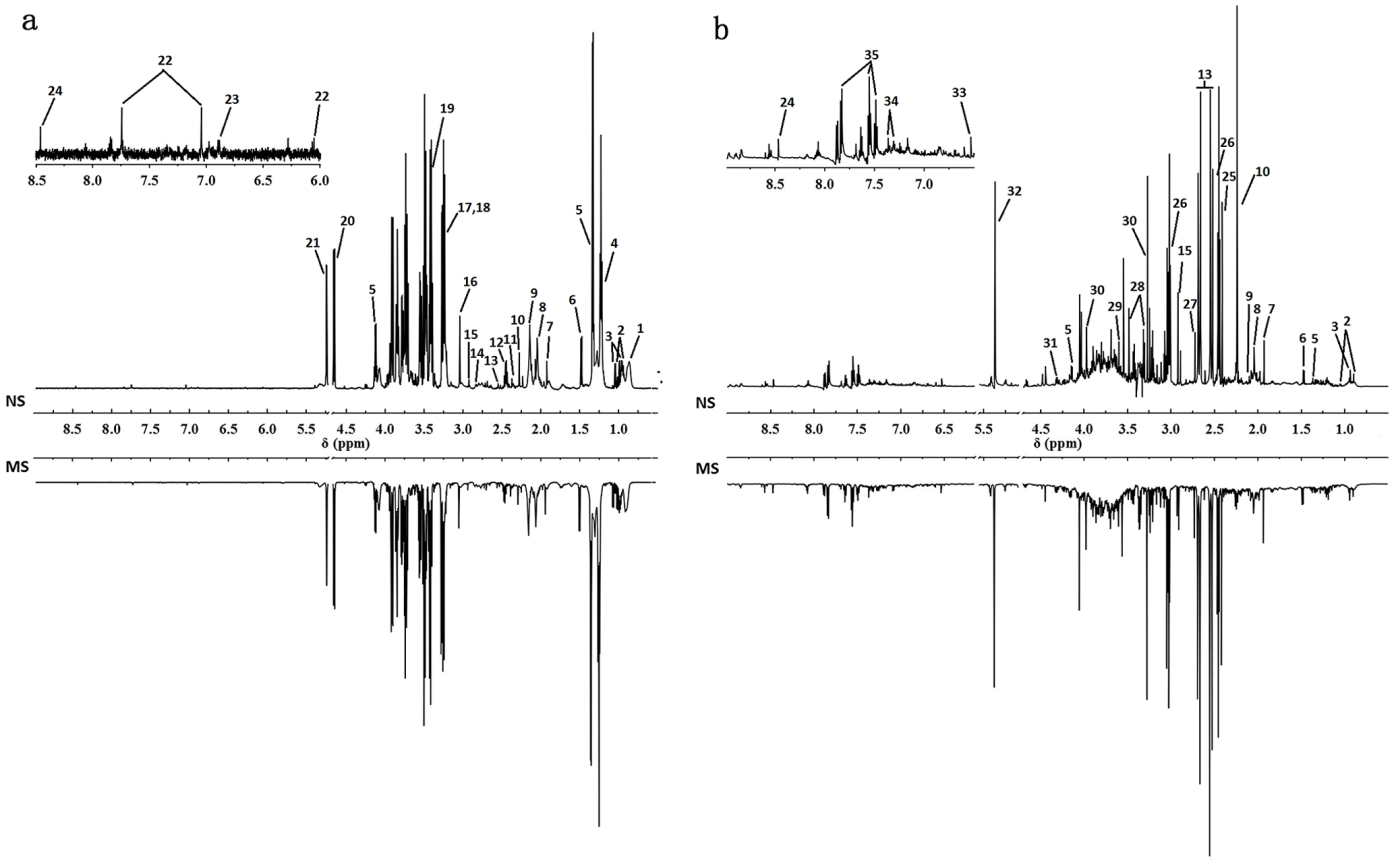
Typical 600 MHz ^1^H-NMR standard spectra of serum and urine collected from NS (a) and MS (b) group rats. A total of 35 metabolites were unambiguously assigned, their chemical shifts and peak multiplicities are given in Table 1.

Typical ^1^H-NMR spectra obtained for serum and urine are shown in Figure 3. The serum spectra contained peaks mainly from lipoproteins, glycoproteins, glucose, amino acids, carboxylic acids such as lactate and β-HB and choline-containing metabolites. Urine spectra were dominated by amines, various organic acids including citrate, succinate, fumarate and lactate and a range of gut microbial-host co-metabolites. The metabolite resonances were assigned according to previous studies [28] and the Chenomx NMR suite (Chenomx Inc., Edmonton, AB, Canada). The identification of metabolites is shown in Table 1.

**Table 1:** ^1^H-NMR data and assignments of the metabolites in rat serum and urine.

Key	Metabolites	Moieties	Chemical shift^a^	Samples^b^
1	Lipid	CH_3_, (CH_2_)_n_, CH_2_-C = C	**0.89(m)**, 1.27(m), 2.0(m)	S
2	Leu/Ile	α CH, β CH_2_, γ CH_3_, δ CH_3_	3.65(d), 1.95(m), **0.94(t), 1.02(d)**	S,U
3	Valine	α CH, β CH_2_, γ CH_3_, δ CH_3_	**0.99(d)**, 3.72(t), 1.96(m), 0.91(d)	S,U
4	β-HB	CH, CH_2_, γ CH_3_, CH_2_	4.16(dt), 2.41(dd), **1.20(d)**, 2.31(dd)	S
5	Lactate	α CH, β CH_3_	**4.11(q), 1.32(d)**	S,U
6	Alanine	α CH, β CH_3_	3.77(q), **1.48(d)**	S,U
7	Acetate	CH_3_	**1.91(s)**	S,U
8	N-acetyl-glycoproteins	CH_3_	**2.04(s)**	S,U
9	O-acetyl-glycoproteins	CH_3_	**2.14(s)**	S,U
10	Acetoacetate	CH_3_	**2.27(s)**	S,U
11	Glutamate	α CH, β CH_2_, γ CH_2_	2.08(m), **2.34(m)**, 3.75(m)	S
12	Glutamine	α CH, β CH_2_, γ CH_2_	2.15(m), **2.44(m)**, 3.77(m)	S
13	Citrate	CH_2_ (1/2), CH_2_ (1/2)	**2.55(d), 2.65(d)**	S,U
14	Aspartic acid	CH_2_	**2.82(dd)**	S
15	TMA	CH_3_	**2.93(s)**	S,U
16	Creatinine	CH_3_, CH_2_	**3.03(s)**, 3.92(s)	S
17	Choline	N(CH_3_)_3_, OCH_2_, NCH_2_	**3.2(s)**, 4.05(t), 3.51(t)	S
18	Phosphocholine	N(CH_3_)_3_, OCH_2_, NCH_2_	**3.22(s)**, 4.21(t), 3.61(t)	S
19	TMAO	CH_3_	**3.27(s)**	S
20	β-Glucose	1-CH	**4.66(d)**	S
21	α-Glucose	1-CH	**5.23(d)**	S
22	Histidine	2-CH, 4-CH, CH_2_	7.75(t), 7.08(d), **6.05(d)**	S
23	Tyrosine	CH, CH	**6.89(dd)**, 7.18(dd)	S
24	Formate	CH	**8.45(s)**	S,U
25	Succinate	CH_3_	**2.41(s)**	U
26	2-oxoglutarate(2-OG)	CH_2_, CH_3_	**2.45(t), 3.01(t)**	U
27	Dimethylamine	CH_3_	**2.72(s)**	U
28	Taurine	S-CH_2_, N-CH_2_	**3.26(t), 3.40(t)**	U
29	Glycine	CH_2_	**3.57(s)**	U
30	Betaine	CH_2_, CH_3_	**3.26(s), 3.93(s)**	U
31	Malate	CH	**4.31(dd)**	U
32	Allantoate	CH	**5.40(s)**	U
33	Fumarate	CH	**6.53(s)**	U
34	Phenylacetylglycine	2,6-CH, 3,5-CH, 7-CH, 10-CH	**7.43(m), 7.37(m),** 3.75(d), 3.68(s)	U
35	Hippurate	CH_2_, CH, CH, CH, NH	3.98(d), **7.56(t), 7.65(t), 7.84(d)**	U

a s, singlet; d, doublet; t, triplet; q, quartet; m, multiplet; dd, doublet of doublets. Bold letters indicated these peaks were visually assigned in Figure 3.

b S, serum; U, urine.

**Figure 4:**
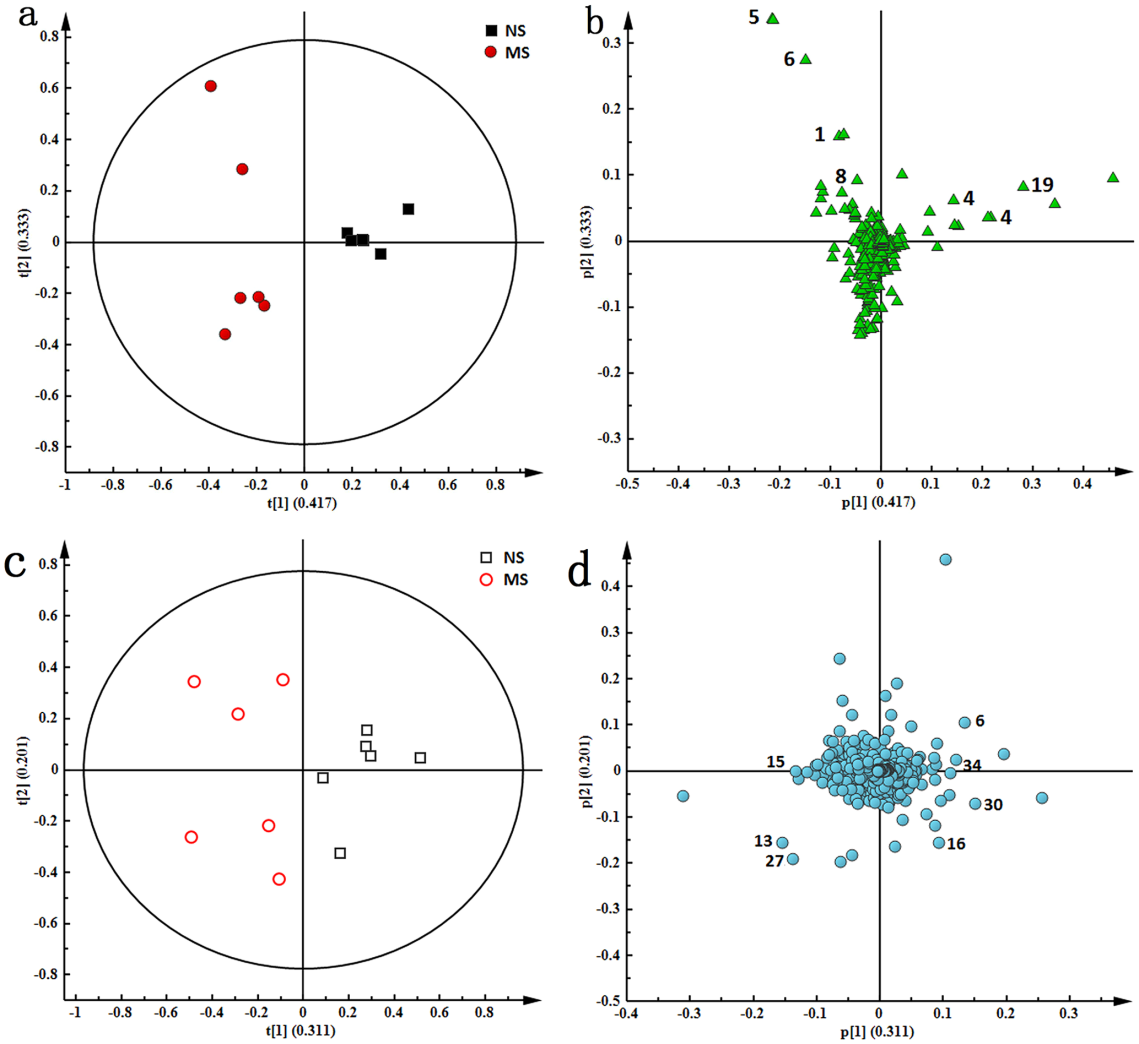
PCA score plots (a, c) and corresponding loading plots (b, d) based on ^1^H-NMR spectra of serum (a, b) and urine (c, d) between NS and MS groups.

The score plots, revealing the inherent clustering of groups of data based on the similarity/dissimilarity of their input coordinates, indicated a clear discrimination between the NS group and the MS group (Figure 4a and Figure 4c). This result mirrored the differences between the groups in the behavioral tests. These data demonstrated that the CUMS model was reliable and consistent, and that metabonomics was a valid approach for the study of depression. The metabolites responsible for the significant separation between the MS group and the NS group were identified in the corresponding loading plots (Figure 4b and Figure 4d). Significant differences in the levels of these metabolites were obtained by independent sample *t*-tests. We observed higher levels of lipid (δ 0.89, m), lactate (δ 1.32, d), alanine (δ 1.48, d), and N-acetyl glycoproteins (δ 2.04, s), and lower levels of β-HB (δ 1.20, d) and TMAO (δ 3.27, s) in the serum of the MS group compared with the NS group. The changes in these endogenous metabolites are considered to be a direct result of the perturbation induced by the CUMS procedure. The PCA score plots and loading plots of urine ^1^H-NMR spectra are shown in Figure 4c and Figure 4d and suggest that the CUMS model was successfully duplicated. The corresponding loading plots demonstrated the metabolites responsible for the variation in the two groups; higher levels of citrate (δ 2.55, 2.65, dd), TMA (δ 2.93, s) and dimethylamine (δ 2.72, s) and lower levels of creatinine (δ 3.03, s) and betaine (δ 3.26, s) were observed in the MS group compared with the NS group.

**Figure 5:**
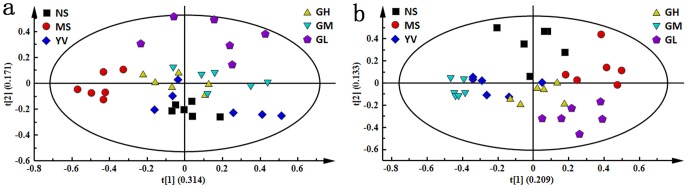
PCA score plots based ^1^H-NMR spectra of serum (a) and urine (b) samples from NS, MS, YV, GH, GM and GL groups.

The PCA score plots present a global view of the response of an organism to stimulations such as stresses or drug administration. To generate an overview of the metabolic response of rats to CUMS exposure and drug administration, the PCA trajectories (Figure 5) of both serum and urine profiles were used. Seen in Figure 5, the NS group was completely separated from the MS group, while the YV, GH and GM groups were all settled around the NS group. Of the genipin-treated groups, the GH group was closest to the NS group, indicating that a high dose of genipin resulted in the best anti-depressive effect.

**Figure 6:**
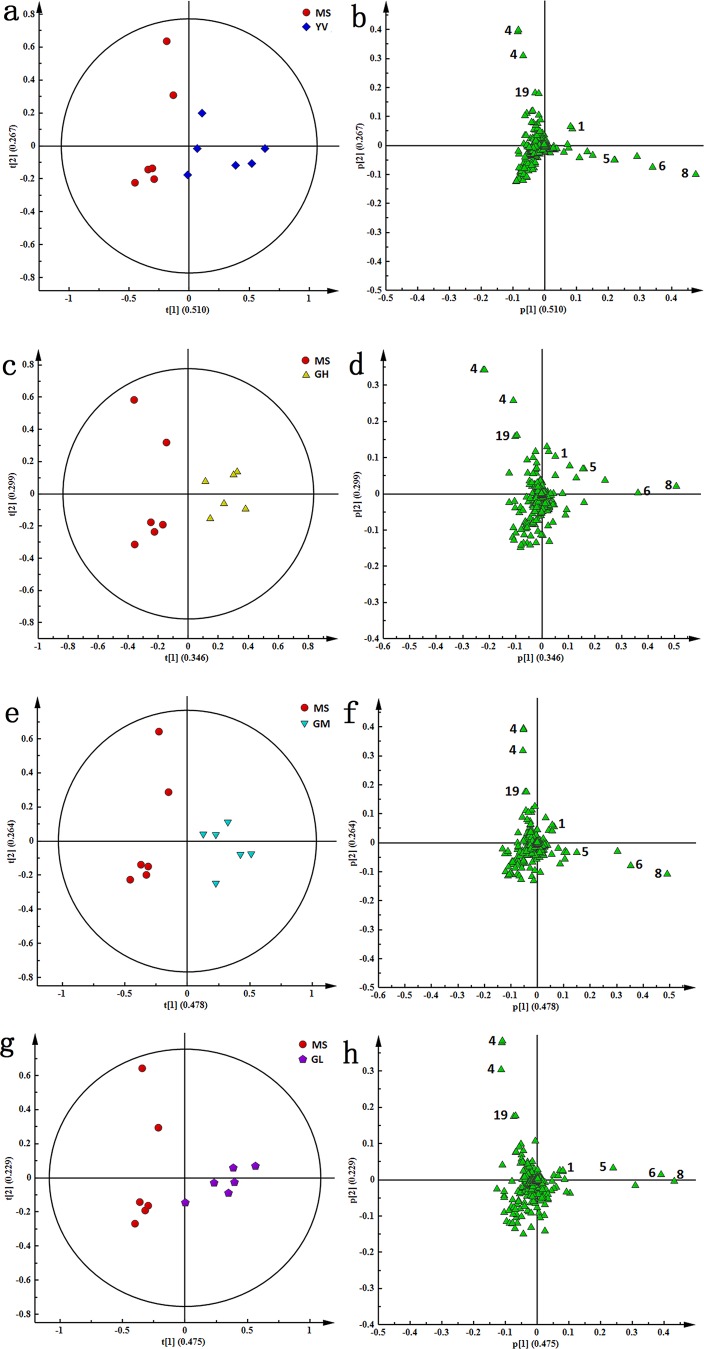
PCA score plots (a, c, e, and g) and corresponding loading plots (b, d, f and h) based on ^1^H-NMR spectra of serum samples between MS group and other treatment groups including YV, GH, GM and GL groups, respectively.

**Figure 7:**
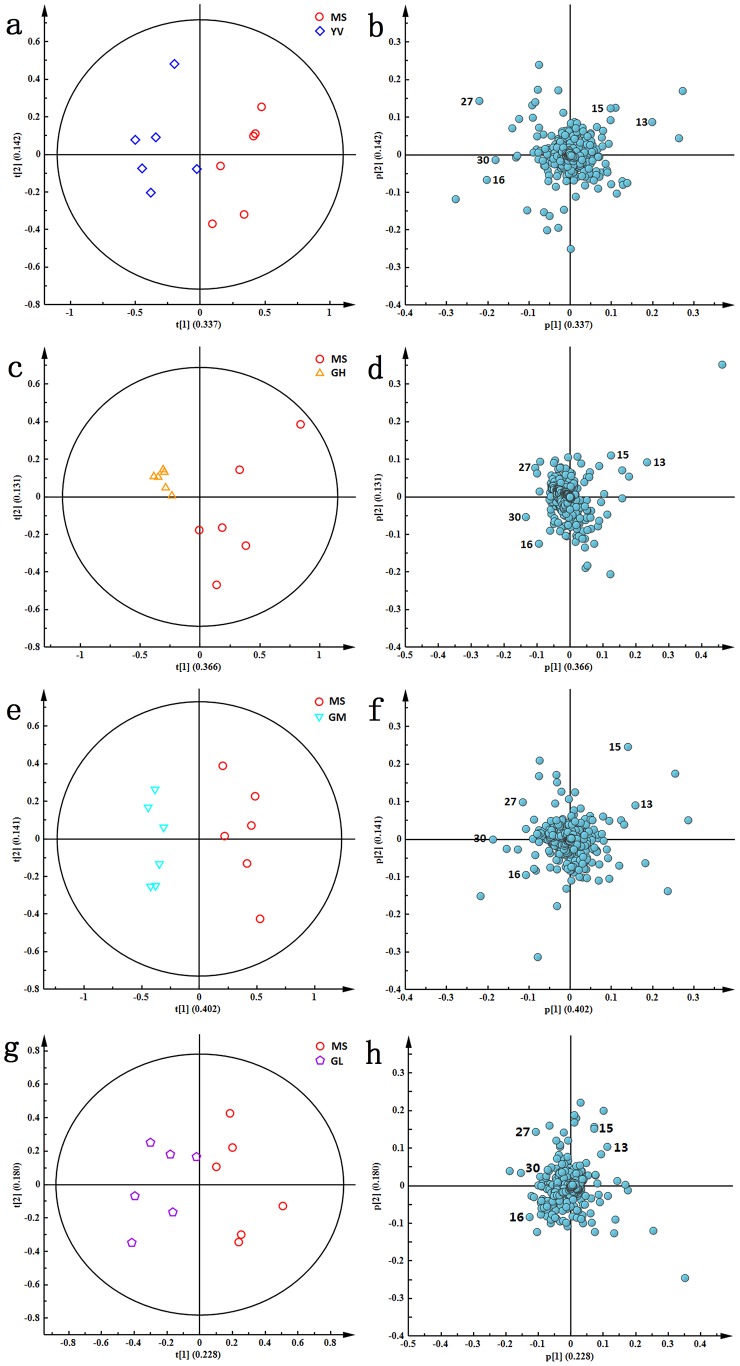
PCA score plots (a, c, e, and g) and corresponding loading plots (b, d, f and h) based on ^1^H-NMR spectra of urine samples between MS group and other treatment groups including YV, GH, GM and GL groups, respectively.

The PCA score plots of both serum and urine comparing NS and MS, MS and YV, MS and GH, MS and GM, MS and GL are shown in Figure 6 and Figure 7. To identify the metabolites contributing to the anti-depressive effects of genipin, PCA loading plots of the MS group and each treatment group (Figure 6 and Figure 7) were analyzed and independent sample *t*-tests were conducted.

### Perturbed metabolic pathways in response of CUMS and Drug Administration

**Figure 8:**
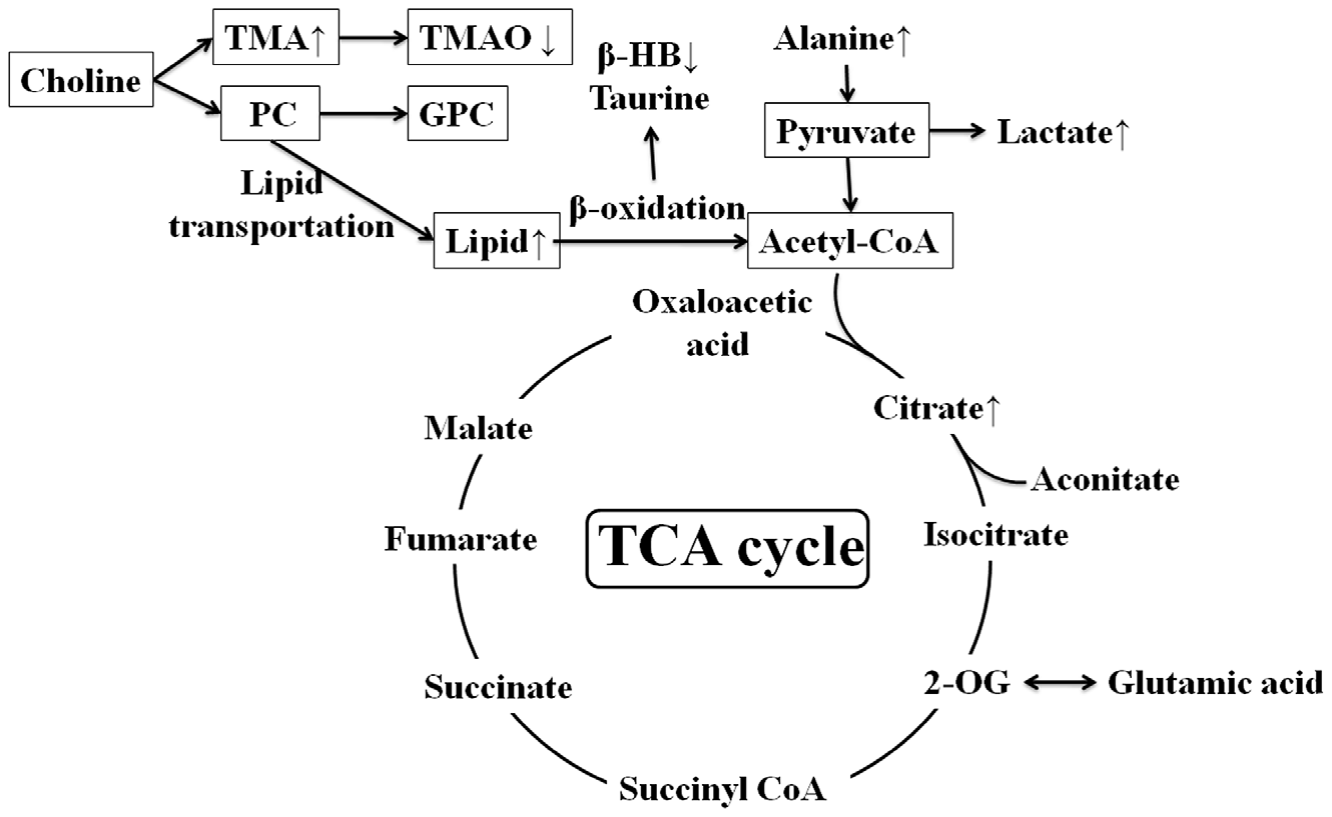
An overview of the metabolic pathways related to the CUMS-induced depression. The ↑ or ↓ was a demonstration of increased or decreased metabolites in serum and urine as compared with the control rats.

In this study, the concentrations of 6 endogenous metabolites in serum (Table 2) and 5 in urine (Table 3) were significantly affected by the CUMS procedure and drug administration. Figure 8 shows the changes in the metabolic pathway induced by a four-week CUMS procedure. Figure 8 suggests that the CUMS procedure mainly disturbed gut microbes, energy metabolism and glycometabolism. After treatment with antidepressant for two weeks, the concentrations of these metabolites showed a tendency to be revised from the level of the CUMS-treated group to reach normal levels in both serum (Table 2) and urine (Table 3). The effect of genipin was similar to that of venlafaxine, indicating a protective effect of genipin against the depressive state induced by the CUMS procedure. A comparison of the anti-depressive effect of different doses of genipin showed that the high dose of genipin (100 mg/kg body weight) most effectively prevented CUMS induced changes in concentration of metabolites.

**Table 2:** Key changed metabolites as potential biomarkers in the rat serum based on ^1^H-NMR loading plots.

Key	Metabolites	NS	YV	GH	GM	GL
1	Lipid	↓*	↓*	↓**	↓*	-
4	β-HB	↑**	↑**	↑**	↑*	-
5	Lactate	↓**	↓**	↓*	↓*	↓*
6	Alanine	↓**	↓**	↓*	↓*	-
8	N-acetyl glycoproteins	↓**	↓**	↓**	↓*	↓*
19	TMAO	↑**	↑**	↑**	↑*	-

↑ or ↓ represent the metabolite increased or decreased as compared with MS group; - represents no significant change compared with MS group;

** and* represent *p*<0.01 or p<0.05 compared with MS group.

**Table 3:** Key changed metabolites as potential biomarkers in the rat urine based on ^1^H-NMR loading plots.

Key	Metabolites	NS	YV	GH	GM	GL
13	Citrate	↓**	↓**	↓**	↓**	↓**
15	TMA	↓**	↓**	↓**	↓**	↓**
16	Creatinine	↑**	↑**	↑**	↑*	-
27	Dimethylamine	↓**	↓**	↓**	↓**	↓*
30	Betaine	↑**	↑**	↑**	↑*	-

↑ or ↓ represent the metabolite increased or decreased as compared with MS group; - represents no significant change compared with MS group;

** and* represent *p*<0.01 or p<0.05 compared with MS group.

Citrate is a dominant product of the tricarboxylic acid cycle (TCA), and the increase in citrate is associated with glycometabolism and energy metabolism. At the same time, the increase in serum lactate, the end-product of glycolysis, indicates the inhibition of glycolysis and the inhibition of gluconeogenesis. This may be related to anaerobic cell respiration, an energy metabolism process that indicates low metabolism efficiency [29]. The increase in serum alanine after the CUMS procedure is likely the result of protein catabolism under stress, with a possible contribution from generally reduced levels of gluconeogenesis [30]. The reduced activity of the TCA cycle could reduce ATP generation in mitochondria and potentially induce fatigue, a symptom frequently observed in depressed patients and animals [31].

TMA is derived from the degradation of choline and carnitine by bacterial enzymes in the intestine [32]. TMA is then normally oxidized to TMAO by the flavin monooxygenase system in the liver [33]. The increase in the concentration of TMA, as well as the decline of dimethylamine in the urine of CUMS-treated rats, may be evidence of a relationship between depression and irritable bowel syndrome [34].

The decrease in serum β-HB, a known ketone body, indicates a reduction of energy production through fatty acid oxidation, which is further proven by increased levels of lipid in the serum. These changes suggest that dysfunction of carbohydrate and energy metabolism may be present in depressive animals [35].

Although most of the broad protein resonances were suppressed by the CPMG pulse sequence, there was a significant elevation of signal intensities of N-acetyl glycoproteins in the MS group animals. N-acetyl glycoproteins are acute-phase reaction glycoproteins detected in serum [36]. Previous studies have shown that depression may be complicated by acute phase responses [37], [38]. In the present study, the increased N-acetyl glycoproteins in the MS group were evidence of an inflammatory response resulting from the depression procedure.

Urinary creatinine level was significantly decreased after the CUMS procedure. Creatinine, a breakdown product of creatine phosphate in muscles, is generally eliminated from the kidneys by glomerular filtration with partial tubular excretion. Because the level of urinary creatinine is constant in healthy animals, the decreased levels of creatinine in CUMS-treated animals might be related to the decrease in muscle mass resulting from stress-induced weight loss. A similar phenomenon has been observed in obesity research [39], indicating there may be a relationship between depression and obesity.

Betaine is an osmolyte and essential methylation agent in the conversion of homocysteine to methionine in the methionine cycle [40]. The decreased levels of betaine in the urine of CUMS-treated rats indicate suppression of the methionine cycle.

After drug administration for two weeks, the effect of CUMS treatment on these metabolites was reversed to varying degrees. In the three genipin groups, the GH group best recovered the regulation of metabolites, suggesting that the anti-depressive effect of genipin is most effective at a dose of 100 mg/kg body weight.

## Conclusions

In conclusion, to investigate the anti-depressant effect of genipin, we used a metabonomic approach based on ^1^H-NMR with multivariate analysis to monitor the metabolic profile of biofluids in rats. The results indicated that the best anti-depressive effect occurred at a genipin dose of 100 mg/kg body weight. Six metabolites in the serum were identified as potential biomarkers: lipid, β-HB, lactate, alanine, N-acetyl glycoproteins and TMAO. Five metabolites in urine were identified: citrate, TMA, creatinine, dimethylamine and betaine. These data indicated that genipin may play an anti-depressant role through regulating gut microbes, energy metabolism and glycometabolism.
